# Expression of extracellular matrix metalloproteinase inducer (EMMPRIN) and its related extracellular matrix degrading enzymes in the endometrium during estrous cycle and early gestation in cattle

**DOI:** 10.1186/1477-7827-8-60

**Published:** 2010-06-11

**Authors:** Birendra Mishra, Keiichiro Kizaki, Katsuo Koshi, Koichi Ushizawa, Toru Takahashi, Misa Hosoe, Takashi Sato, Akira Ito, Kazuyoshi Hashizume

**Affiliations:** 1 Laboratory of Veterinary Physiology, Department of Veterinary Medicine, Iwate University, Ueda 3-18-8, Morioka, Iwate 020-8550, Japan; 2 United Graduate School of Veterinary Science, Gifu University, Japan; 3 Department of Developmental Biology, National Institute of Agrobiological Sciences, Ikenodai 2, Tsukuba, Ibaraki 305-8602, Japan; 4 Department of Biochemistry and Molecular Biology, Tokyo University of Pharmacy and Life Sciences, Hachioji, Tokyo 192-0392, Japan

## Abstract

**Background:**

Extracellular matrix metalloproteinase inducer (EMMPRIN) regulates several biological functions involving the modulation of cell behaviors via cell-cell and cell-matrix interactions. According to its diverse functions, we hypothesized that EMMPRIN may play an important role in endometrial remodeling and establishment of pregnancy in cow.

**Methods:**

In this study, endometrial tissues from the cyclic cows during before ovulation, after ovulation and middle of estrous cycle; and pregnant endometrial tissues from Day 19 to 35 of gestation have been used. Expression of mRNA was analyzed by RT-PCR, qPCR and in situ hybridization whereas protein expression by immunohistochemistry and western blot analysis.

**Results:**

EMMPRIN mRNA was expressed in both cyclic and pregnant endometrium and significantly higher in the endometrium at Day 35 of gestation than the cyclic endometrium. In Western blot analysis, an approximately 65 kDa band was detected in the endometrium, and approximately 51 kDa in the cultured bovine epithelial cells and BT-1 cells, respectively. Both in situ hybridization and immunohistochemistry data showed that EMMPRIN was primarily expressed in luminal and glandular epithelium with strong staining on Day 19 conceptus. At Day 19 of gestation, expression of EMMPRIN mRNA on luminal epithelium was decreased than that observed at middle of estrous cycle, however, on Day 30 of gestation, slightly increased expression was found at the site of placentation. Expression of matrix metalloproteinase-2 (MMP-2) and MMP-14 mRNA were mainly detected in stroma and their expression also decreased at Day 19 of gestation however it was also expressed at the site of placentation at Day 30 of gestation as observed for EMMPRIN. Expression of MMP-1 or -9 mRNA was very low and was below the detection limit in the cyclic and pregnant endometrium.

**Conclusion:**

EMMPRIN from the luminal epithelium may regulate the expression of stromal MMP-2 and -14 suggesting its crucial role in adhesion and fusion of embryo to luminal epithelium by directly itself through physiological tissues remodeling and developmental process, and/or stimulating MMPs to compensate endometrial functions.

## Background

The bovine endometrium is a highly complex and dynamic tissue composed of the caruncular endometrium, which acts as the implantation site and consists of a thickened subepithelial stroma, and another region termed the intercaruncular endometrium located between the caruncles containing glandular regions [1]. There are certain requirements for successful implantation in ruminants. Firstly, the endometrium needs to be prepared for this event during the estrous cycle, and especially around implantation; and secondly, the trophoblast must be able to fuse with the endometrial epithelium in the caruncle region. In this regard, endometrial remodeling during the estrous cycle and early gestation remains crucial for the establishment of pregnancy. In human, sloughing of the functional layer of the endometrium and subsequent healing occur, which are highly regulated by matrix metalloproteinases (MMP) [2,3]; however, this phenomenon does not occur in ruminants, although the endometrium undergoes dynamic changes in its mRNA profile accompanied by morphological changes during the estrous cycle [4,5]. During the estrous cycle and the establishment of pregnancy, endometrial cells undergo rapid growth and differentiation, extracellular matrix (ECM) break down and remodeling [6,7]. Around implantation, a number of molecules are expressed at the embryo-maternal interface including interferon-tau, cytokines, growth factors, hormones, and MMP [8-11]. These changes in the endometrium are partly modulated by the expression of the MMP system [12-14], a disintegrin and metalloproteinase with thrombospondin motif (ADAMTS)-1 [15,16], and extracellular matrix metalloproteinase inducer (EMMPRIN) [17] in coordination with ovarian steroids [18,19].

EMMPRIN (also known as Basigin or CD147) is a multidomains, multifunctional glycoprotein located on the cell surface in physiological and pathological conditions including tumor cells [20-24]. EMMPRIN is associated with cell growth and adhesion [25], angiogenesis [26,27], chaperone functions [28,29], immune cell activation [30], proMMP-1 activation [31], MMP induction [32,33], and ECM degradation and remodeling [34]. EMMPRIN is highly associated with the menstrual cycle [35,36], estrous cycle, and pregnancy in mice [17] and rats [37], as well as ovulation and luteogenesis [38]. In cow, EMMPRIN may be involved in endometrial remodeling for implantation same as those in other species because recent reports showed that MMP and ECM-related molecules including EMMPRIN expressed in bovine endometrium and placenta [10,14]. These previous reports strongly suggest that EMMPRIN takes a vigorous role for implantation process in various species irrespectively. However, the expression of EMMPRIN and its role in implantation are still obscure in cow. At present, there has been no report on the expression and role of EMMPRIN in the bovine endometrium. Therefore, the aim of our study was to examine the expression and cellular localization of EMMPRIN along with MMP-1, -2, -9 and -14 in the bovine endometrium during the estrous cycle and early gestation, secondly, how EMMPRIN regulates MMPs at the feto-maternal interface during this critical period.

## Methods

### Animals and tissue collection

Endometrial tissues were collected from Japanese Black cows at different functional stages of estrous cycle and early gestation for RT-PCR, quantitative RT-PCR (qPCR), in situ hybridization, Western blotting and immunohistochemistry according to the objectives of this study. The physiological status of the cyclic endometrial tissues was estimated based on ovarian morphology as described previously [39]. Namely, the approximate day of the estrous cycle was determined in four distinct sequential stages by the appearance of the corpus luteum and the size of the follicles: stage I - days 1 to 4; stage II - days 5 to 10; stage III - days 11 to 17; stage IV-days 18 to 20. In the present study, we reorganized the estrous cycle into three stages as follows: stage after ovulation (AOV) - days 1 to 4 (n = 3), middle of estrous cycle (MEC) stage - days 5 to 17 (n = 3), and stage before ovulation (BOV) - days 18 to 20 (n = 3). Pregnant tissues were collected during the periimplantation period, Day 19 (totally n = 3, two cows on day 19 and one cow on day 20) and post implantation period; Day 30 (totally n = 3, including one cow on day 28 and two cows on day 30) and Day 35 (totally n = 3, two cows on day 35 and one cow on day 37). Pregnancy was derived by artificial insemination (AI) (the day of insemination was designated as Day 0 of gestation). Histological studies were performed using endometrium from estrous cycle (BOV, AOV, MEC), and Day 19 and Day 30 of gestation. These tissues were fixed in 4% paraformaldehyde-phosphate buffered saline (PBS, pH 7.4) and stored at 4°C. Some pregnant uteri were perfused with 4% (w/v) paraformaldehyde PBS on early gestation and all tissues for histological examination were managed according to the previous report [40]. RT-PCR and Western blot analysis were done using middle of estrous cycle. qPCR was performed using cyclic endometrial tissues and Day 35 of pregnant endometrial and fetal tissues. Briefly, immediately after collection, all tissues were snap frozen in liquid nitrogen and stored at -80°C until RNA extraction. Some uterine tissues were collected for histological studies as mentioned above. All animal procedures were carried out in accordance with the guidelines and ethics set out by the Animal Care and Use Committee of the Iwate University and the National Institute of Agrobiological Sciences, Japan.

### RNA extraction and RT-PCR

Total RNA was individually isolated using TRIzol (Invitrogen, Carlsbad, CA, USA) according to the manufacturer's instructions. The yield of total RNA was quantified by measuring the absorbance at 260 nm (A260). RNA quality was determined by measuring the A260/A280 ratio using a NanoDrop Spectrophotometer (ND-1000, Wilmington, DE, USA) and by 1% agarose gel electrophoresis. Two micrograms of total RNA was reverse transcribed into cDNA using random primers with a high capacity reverse transcriptase kit (Applied Biosystems, Foster City, CA, USA) according to the manufacturer's instructions. The RT cycle comprised 25°C for 10 minutes, 37°C for 120 minutes, and 85°C for 5 seconds in the thermal cycler, and cDNA were stored at -20°C. The PCR primers were designed using the Primer-3 software [41] based on bovine sequences (Table 1). The PCR reaction was performed at 95°C for 30 seconds for denaturing, 60°C for 30 seconds for annealing, and at 72°C for 1 minute for extension and was repeated for 30 cycles. The PCR products were subjected to electrophoresis using 2% gel and stained with ethidium bromide solution. The amplified products were sub-cloned into the pGEM-T Easy vector (Promega, Madison, WI, USA) and sequenced using the Big dye terminator cycle sequencing kit and an automated sequencer (Applied Biosystems).

**Table 1:** Oligonucleotide primers used for standard RT-PCR analysis

Gene	Primer	Sequence	Position
EMMPRIN	Forward	TCCAAAACACGACTCACCTGTG	153-174
(NM_001075371)	Reverse	GCTTCCGCCTCTTCTCGTAGAT	753-732
MMP-1	Forward	TGCTCATGCTTTTCAACCAG	554-574
(NM_174112)	Reverse	TCCGGGAAAGTCTTCTGCTA	1274-1254
MMP-2	Forward	CAGACAGTGGATGATGCCTTCG	596-618
(NM_174745)	Reverse	GCGGCCTGTGTCTGTGCAGC	940-920
MMP-9	Forward	TGGACATCTTCGACGCCATC	1574-1593
(NM_174174)	Reverse	CGAACCTCCAGAAGGTCTGC	1927-1908
MMP-14	Forward	GGCTGATGCAGACACCATGAA	290-310
(NM_174390) GAPDH (NM_001034034)	Reverse Forward Reverse	GGATCGTTGGAATGCTCAAGG CCTTCATTGACCTTCACTACATGGTCTA GCTGTAGCCAAATTCATTGTCGTACCA	811-791 173-201 1029-1002

### Quantitative real-time RT-PCR (qPCR)

The gene expression of EMMPRIN, MMP-1, -2, -9, and -14 was confirmed at each stage of the estrous cycle and Day 35 of gestation by qPCR using the SYBR Green assay (Applied Biosystems) as described previously [42]. In the SYBR Green assay, primer pairs were designed using the Primer Express software program (Applied Biosystems) (Table 2). The standard curves for each gene were generated by serial dilution of a plasmid containing EMMPRIN, MMP-1, -2, -9, and -14 cDNA in order to quantify the mRNA concentrations. We confirmed the melting curve for detecting the SYBR Green-based objective amplicon because SYBR Green also detects double-stranded DNA including primer dimers, contaminating DNA, and PCR products from misannealed primers. Contaminating DNA or primer dimers appear as peaks separate from the desired amplicon peak. The expression ratio of each gene to GAPDH mRNA was calculated to adjust for variations in the RT-PCR reaction.

**Table 2:** Oligonucleotide primers used for Real-time RT-PCR analysis

Gene	Primer	Sequence	Position
EMMPRIN	Forward	CGGAGTATGAGGTGGACTCAGAA	265-288
(NM_001075371)	Reverse	GCTCCGGAAGGAAGATGCA	327-308
MMP-1	Forward	TGGACCAGCAATTTCCAAGAT	618-639
(NM_174112)	Reverse	TCCAAGGGAATGGCCAAA	686-668
MMP-2	Forward	GGAGAGGCTGACATCATGATCA	677-699
(NM_174745)	Reverse	CCCGTCTTTGCCATCAAAA	751-770
MMP-9	Forward	CATTTCTTCAAGGCTGGGAAGTA	1615-1638
(NM_174174)	Reverse	GCGCAGGCCACTTGCT	1708-1692
MMP-14	Forward	TGCCGAGCCTTGGACTGT	704-722
(NM_174390) GAPDH (NM_001034034)	Reverse Forward Reverse	GCCACCAGAAAGATGTCATTCC AAGGCCATCACCATCTTCCA CCACCACATACTCAGCACCAGCAT	763-741 280-300 355-331

### In situ hybridization analysis

The bovine cDNA were used as templates for hybridization probe synthesis. Digoxigenin (DIG)-labelled antisense and sense-complementary RNA probes were prepared using DIG RNA labelling kits (Roche, Diagnostic GmbH, Mannheim, Germany) and cloned cDNA in plasmids as described previously [42]. Formalin-fixed tissue was positioned for paraffin embedding using the methods described previously [42]. Briefly, the tissues were treated with a series of alcohols (from 70% to absolute alcohol), xylene, and paraffin. The endometrial tissues were then cut into 7 μm-thick sections, and *in situ *hybridization was performed using an automated Ventana HX System Discovery with a RiboMapKit and a BlueMapKit (Ventana, Tucson, AZ, USA). Briefly, the sections were hybridized with DIG-labelled probes in RiboHybe (Ventana) hybridization solution at 61°C for EMMPRIN, MMP-1, -2, -9, and -14 for 6 hours, before being washed 3 times for 6 minutes in RiboWash (Ventana) at 65°C and fixed in RiboFix (Ventana) at 37°C for 10 min. The hybridization signal of each gene was detected using rabbit monoclonal anti-digoxin biotin conjugates (Sigma, Saint Louis, MO, USA) and an AmpMapKit (Roche/Ventana). Counterstaining was performed with nuclear fast red (Roche/Ventana). After preparation, the hybridized slides were observed with a Leica DMRE HC microscope (Leica Microsystems, Wetzlar, Germany) equipped with a DS-Fi1 camera and a DS-L2 control unit (Nikon, Tokyo, Japan).

### Immunohistochemistry

For immunohistochemical analysis of EMMPRIN, formalin fixed and paraffin embedded endometrial tissues were cut into 7 μm-thick sections as mentioned for the *in situ *hybridization. Briefly, the sections were permeabilized with proteinase K and incubated with a primary EMMPRIN antibody at a dilution of 1:1000 in antibody diluents (Ventana). The rabbit polyclonal anti-human EMMPRIN antibody was developed using three different synthetic peptides, EM1: ^42^SLNDSATEVTGHRWLK^57^, EM6: ^138^AWYKITDSEDKALMN^152^, and EM9: ^170^HIENLNMEADPGQYR^184^. Anti-EM6 antibody, the amino acid sequence of which exists in the second loop domain of human EMMPRIN (Accession number: AB072923) [20] was used in the present study because all three antisera recognized EMMPRIN molecules in SKGII cell lysates but other two antisera recognized some extra bands. Antiserum was further purified by affinity chromatography, and then the affinity-purified antibody was used for immunohistochemistry and Western blot analysis [43].

The sections were then washed and incubated with an anti-rabbit IgG-Biotin conjugate (Sigma) for 1 hour. Immunoreactive signals were detected using streptavidin-horseradish peroxidase (HRP) and diaminobenzidine (DabMapKit, Ventana). In the controls, the normal rabbit sera (NRS; DAKO, CA, USA) were used. Counterstaining was then performed with haematoxylin and bluing reagent (saturated lithium carbonate solution). After treatment, the sections were observed under a microscope equipped as described above.

### Western blotting for EMMPRIN

Western blotting was performed as described previously [44]. Bovine endometrial tissues during estrous cycle were homogenized in a lysis buffer containing 50 mM Tris (pH 7.5), 150 mM NaCl, 0.1% Triton X-100, and the protease inhibitor cocktail Complete, Mini (Roche Diagnostic GmbH). Supernatant of tissues lysate was collected followed by centrifugation at 15,800 × g for 15 minutes at 4°C. Bovine endometrial stromal cells (BES) [45], epithelial cells (BEE, kindly supplied by Dr. Iga) [46], and trophoblastic cells (BT-1), which were derived from bovine blastocysts [47], were maintained in DMEM/F12 with 10% fetal bovine serum until full confluence [45]. The confluenced cells were then washed with serum free media followed by PBS and homogenized in a lysis buffer. After centrifugation at 15,800 × g for 15 minutes at 4°C, the supernatant was collected and preserved for further analysis. The concentration of total protein of both tissues and cell lysates was analyzed using the Quick Start Bradford protein assay kit (Bio-Rad Laboratories, Hercules, CA, USA). Human uterine cervical carcinoma SKG-II cell lysates were used as a positive control [43]. The proteins (28 μg) of each cell lysate were separated by 10% sodium dodecyl sulfate polyacrylamide gel electrophoresis and then transferred to PVDF membranes (Immobilon-P, Millipore Corporation, Bedford, MA, USA). The membranes were first blocked with 5% skimmed milk and then treated with rabbit anti-EMMPRIN antibody as mentioned above at a dilution of 1:1000, before being incubation with alkaline phosphatase-conjugated goat anti-rabbit IgG (Sigma). An alkaline phosphatase detection system (Bio-Rad Laboratories) was used to detect immunoreactive EMMPRIN.

### Statistical analysis

All values are presented as the mean ± SEM. qPCR was duplicated in one animal sample. Statistical analysis was performed by JMP software (SAS Institute Inc, Cary, NC, USA) using one-way ANOVA followed by the Turkey-Kramer test. Different letters indicate significant variation among the statuses of the tissues.

## Results

### Expression and localization of EMMPRIN mRNA in the bovine endometrium

**Figure 1:**
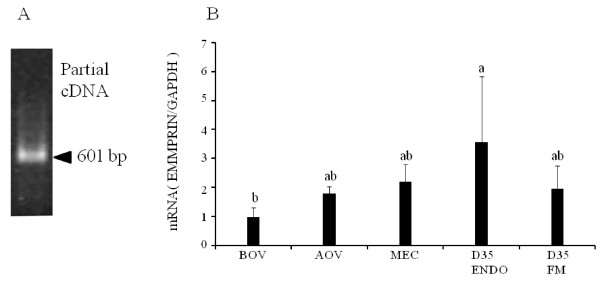
**Expression of EMMPRIN mRNA in the bovine endometrium during the estrous cycle and at Day 35 of gestation**. Partial cDNA was amplified and a 601 bp band was detected as bovine EMMPRIN by RT-PCR (A). Quantitative analysis of EMMPRIN mRNA was performed by qPCR and normalized to GAPDH mRNA expression (B). Bar graph showing the mean ± SD. Data labeled with different letters are significantly different from each other (P < 0.05). bp, base pair; BOV, before ovulation; AOV, after ovulation; MEC, middle of estrous cycle; D35 ENDO, endometrium at Day 35 of gestation, D35 FM, fetal membrane at Day 35 of gestation.

A partial cDNA for bovine EMMPRIN was amplified by RT-PCR from endometrial tissue after being isolated in the middle of the estrous cycle (Figure 1A). A 601 bp cDNA fragment was obtained and identified as the bovine EMMPRIN sequence (NCBI reference sequence NM_001075371).

The expression of EMMPRIN mRNA was analyzed by qPCR during the estrous cycle and at Day 35 of pregnancy (Figure 1B). In the cyclic endometrium, EMMPRIN mRNA expression was slightly increased after ovulation and in the middle of the estrous cycle compared with before ovulation and was significantly higher in the endometrium at Day 35 of gestation. The fetal membrane at Day 35 of gestation also expressed EMMPRIN mRNA but the intensity of expression was slightly lower than that of the endometrium.

**Figure 2:**
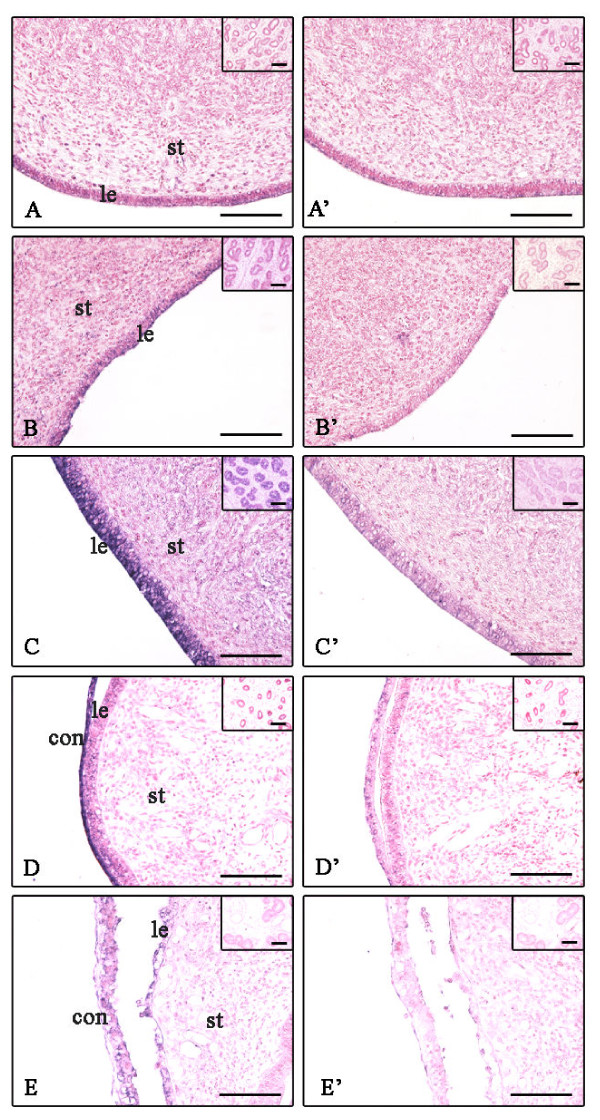
**Localization of EMMPRIN mRNA in the endometrial tissues during the estrous cycle and early gestation**. Seven-micrometer sections of the endometrium were hybridized with DIG labeled antisense cRNA probes (A-E) and sense cRNA probes (A'-E'). A, A': before ovulation (BOV); B, B': after ovulation (AOV); C, C': middle of estrous cycle (MEC); D, D': Day 19 of gestation; E, E': Day 30 of gestation. Superimposed figures showed the glandular region. le, luminal epithelium; st, stroma; con, conceptus. Scale bar = 20 μm (objective; 20×).

The localization of EMMPRIN mRNA was detected in the bovine endometrium during the estrous cycle, at Day 19 and 30 of gestation by *in situ *hybridization analysis (Figure 2, Table 3). Anti-sense RNA probes (Figure 2A-E) for EMMPRIN specifically detected mRNA transcripts in the endometrium, but no significant signals were detected by sense probes (Figure 2A'-E') at any stage of the estrous cycle and early gestation. In the endometrium before ovulation (BOV), EMMPRIN mRNA expression remained below the detection limit (Figure 2A). After ovulation (AOV), it was moderately expressed in luminal epithelium but slightly in stroma and glandular epithelium (Figure 2B); on the other hand, it was strongly expressed in the luminal and glandular epithelia but slightly expressed in the stroma in the middle of the estrous cycle (MEC) (Figure 2C). During early gestation (Day 19), EMMPRIN mRNA was slightly expressed in the luminal and glandular epithelia but remained below the detection limit in the stroma; whereas, strong expression was found in the conceptus (Figure 2D). At Day 30 of gestation, moderately expressed at the site of placentation (Figure 2E).

**Table 3:** Summary of localization of EMMPRIN mRNA and protein expression in the endometrial tissues

	Tissues	BOV	AOV	MEC	Day 19	Day 30
**EMMPRIN**	Stroma	-	+	+	-	-
**mRNA**	LE	-	+ +	+ + +	+	+ +
	GE	-	+	+ + +	+	-
**EMMPRIN**	Stroma	+	+ +	+ +	-/+ + +	NA
**Protein**	LE GE	+ +	+ + +	+ + + + + +	+ + + + +	NA NA

BOV, before ovulation; AOV, after ovulation; MEC, middle of estrous cycle; Day 19, day 19 of gestation; LE, luminal epithelium; GE, glandular epithelium; St, stroma; -, below detection limit; +, slight expression; ++, moderate expression; +++, strong expression; -/+++, upper stroma is below detection limit and lower stroma has strong expression; NA, not analyzed

### Expression and localization of EMMPRIN protein in the bovine endometrium

**Figure 3:**
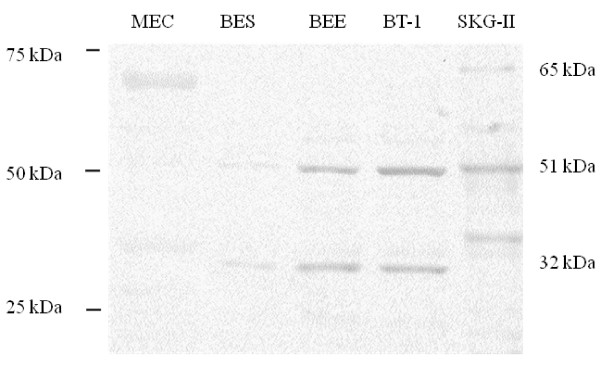
**Expression of EMMPRIN protein in the bovine endometrial tissues by Western blot analysis**. The proteins of bovine endometrial tissues in the middle of the estrous cycle (MEC), endometrial stromal cells (BES), epithelial cells (BEE), trophoblastic cells (BT-1), and human uterine cervical carcinoma cell (SKG-II) were resolved by SDS-PAGE. Molecular weight markers are indicated on the left and protein bands are shown on the right. Results shown are representative of three independent experiments.

In Western blot analysis, three immunoreactive bands were detected with anti-human EMMPRIN antibody in extracts from the bovine endometrium, cultured bovine endometrial stromal cells, epithelial cells, and trophoblastic cells (BT-1), with human uterine cervical carcinoma SKG-II cells used as a positive control (Figure 3). An approximately 65 kDa band was clearly detected and faint 35 kDa was recognized in the MEC endometrium, respectively; however, slight expression was detected at other stages of the estrous cycle (data not shown). On the other hand, the intense band migrated approximately 51 and 32 kDa in the cultured bovine epithelial cells and BT-1 cells, respectively; whereas, a 32 kDa band was detected weakly in the stromal cells.

**Figure 4:**
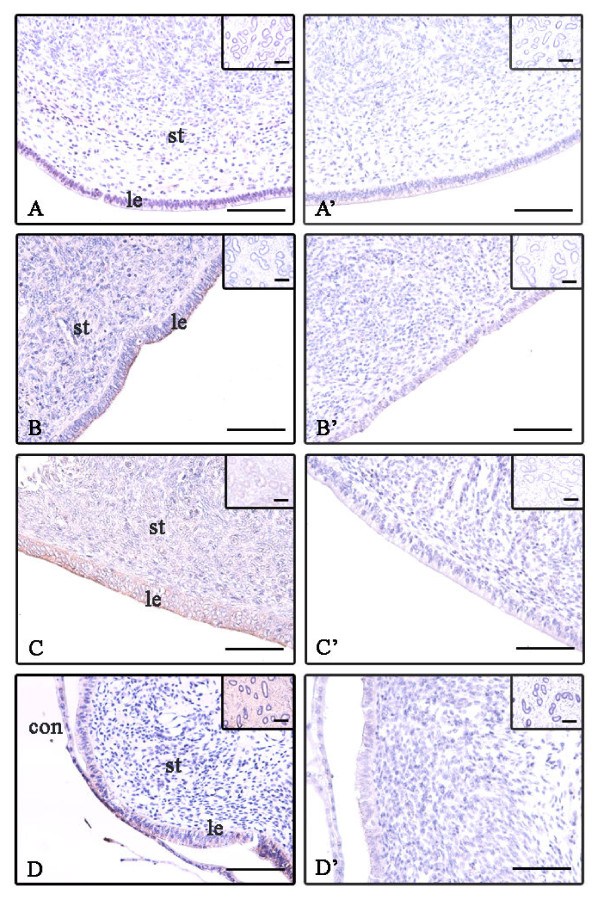
**Localization of EMMPRIN protein in the endometrial tissues throughout the estrous cycle and on Day 19 of gestation according to immunohistochemistry analysis**. Seven-micrometer sections of endometrium were immunostained with anti-EMMPRIN antibody (A-D) and normal rabbit serum (A'-D'). A, A': before ovulation (BOV); B, B': after ovulation (AOV); C, C': middle of estrous cycle (MEC); D, D': Day 19 of gestation. Abbreviations are same as figure 2. Scale bar = 20 μm (objective; 20×).

To confirm EMMPRIN protein localization in the endometrium, we performed immunohistochemical analysis (Figure 4, Table 3). Immunoreactive EMMRIN protein was detected slightly in the stroma, luminal and glandular epithelia in the endometrium at BOV (Figure 4A); whereas, moderate immunostaining was observed in the luminal epithelium and stroma but only slight staining was seen in the glandular epithelium at AOV (Figure 4B). At MEC, strong staining was detected in the luminal and glandular epithelia but moderate staining was found in the stroma (Figure 4C). During early gestation (Day 19), moderate staining of EMMPRIN in luminal epithelium, intense staining was detected in the gland and stroma surrounding the glands and in the conceptus; whereas, protein signals were absent in the stroma under the epithelium (Figure 4D).

### Expression and localization of gelatinases (MMP-2 and MMP-9 mRNA)

**Figure 5:**
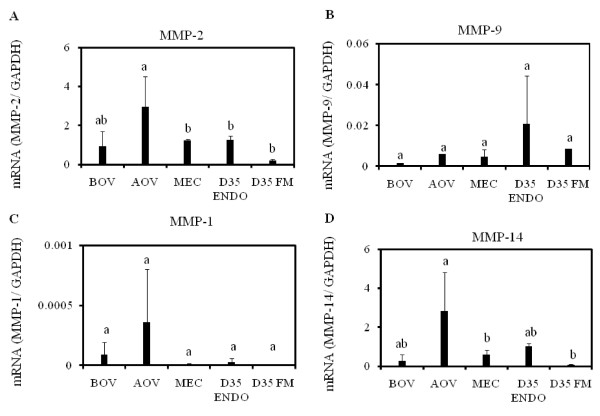
**Expression profile of MMP mRNA in the bovine endometrium during the estrous cycle and at Day 35 of gestation**. Quantitative analysis of MMP mRNA was performed by qPCR and normalized to the expression of GAPDH mRNA. MMP-2 mRNA (A), MMP-9 mRNA (B), MMP-1 mRNA (C), and MMP-14 mRNA (D). Bar graph showing the mean ± SD. Data labeled with different letters are significantly different from each other (P < 0.05). Abbreviations are same as Figure 1.

**Figure 6:**
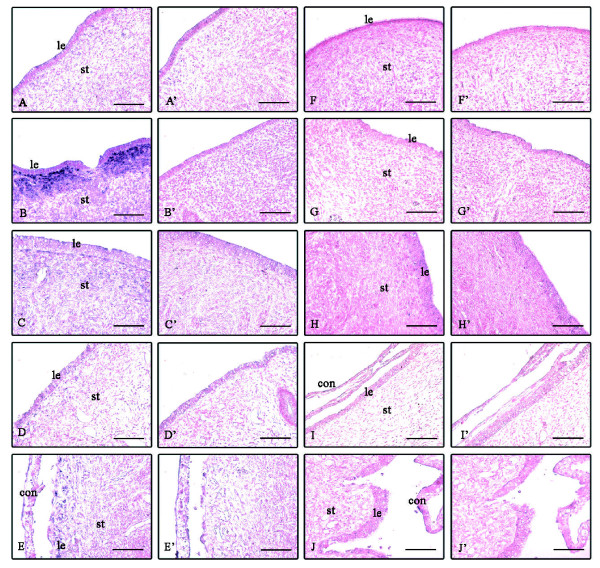
**Localization of MMP-2 and MMP-9 mRNA in the endometrial tissues during the estrous cycle and early gestation**. Seven-micrometer sections of the endometrium were hybridized with DIG labeled antisense cRNA probes of MMP-2 (A-E) and sense cRNA probes (A'-E'). A, A': before ovulation (BOV); B, B': after ovulation (AOV); C, C': middle of estrous cycle (MEC); D, D': Day 19 of gestation; E, E': Day 30 of gestation. MMP-9 mRNA antisense (F-J) and sense cRNA probes (F'-J'). F, F': BOV; G, G': AOV; H, H': MEC; I, I': Day 19; J, J': Day 30 of gestation. Abbreviations are same as Figure 2. Scale bar = 20 μm (objective; 20×).

Expression of MMP-2 and -9 mRNA were detected in the endometrium during the estrous cycle and at Day 35 of gestation by qPCR. MMP-2 mRNA was significantly higher at after ovulation and decreased at mid-cycle and Day 35 of gestation; whereas, slight expression was found in the fetal membrane (Figure 5A). *In situ *hybridization data showed that MMP-2 mRNA was below the detection limit at BOV (Figure 6A), strongly localized in the stroma AOV (Figure 6B), and moderately during the middle of the cycle (Figure 6C). At Day 19 of gestation, MMP-2 was slightly expressed in the stroma (Figure 6D) but moderately at the site of placentation at Day 30 of gestation (Figure 6E). The expression levels of MMP-9 mRNA was very low (Figure 5B) whereas its localization remained below the detection limit at each stage in the cyclic and pregnant endometrium (Figure 6F-J). The localization of MMP-2 and -9 mRNA has summarized in Table 4.

**Table 4:** Summary of localization of MMP mRNA expression in the endometrial tissues

Gene	Tissues	BOV	AOV	MEC	Day 19	Day 30
**MMP-1**	Stroma	-	-	-	-	-
	LE	-	-	-	-	-
	GE	-	-	-	-	-
**MMP-2**	Stroma	-	+ + +	+ +	+	+ +
	LE	-	-	-	-	+
	GE	-	-	-	-	-
**MMP-9**	Stroma	-	-	-	-	-
	LE	-	-	-	-	-
	GE	-	-	-	-	-
**MMP-14**	Stroma	+	+ + +	+ +	+	+ +
	LE	-	-	+	+	+ +
	GE	-	-	-	-	-

All indicators are same as Table 3

### Expression and localization of MMP-1 and MMP-14 mRNA

**Figure 7:**
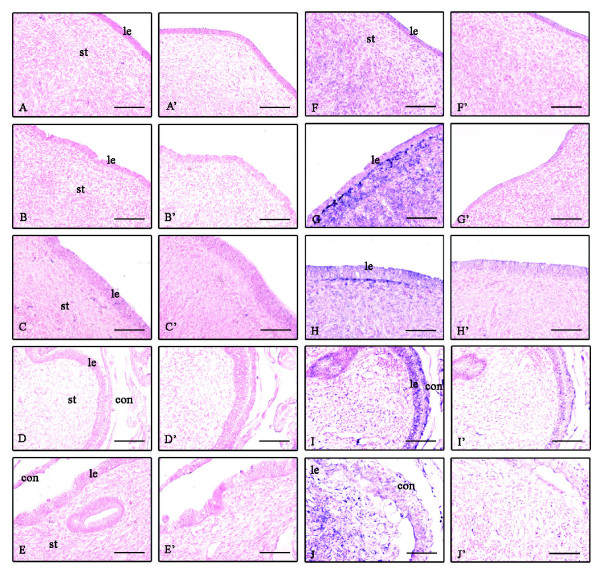
**Localization of MMP-1 and MMP-14 mRNA in the endometrial tissues during the estrous cycle and early gestation**. Seven-micrometer sections of the endometrium were hybridized with DIG labeled antisense cRNA probes of MMP-1 (A-E) and sense cRNA probes (A'-E'). A, A': before ovulation (BOV); B, B': after ovulation (AOV); C, C': middle of estrous cycle (MEC); D, D': Day 19 of gestation; E, E': Day 30 of gestation. MMP-14 mRNA antisense (F-J) and sense cRNA probes (F'-J'). F, F': BOV; G, G': AOV; H, H': MEC; I, I': Day 19; J, J': Day 30 of gestation. Abbreviations are same as Figure 2. Scale bar = 20 μm (objective; 20×).

Expression of MMP-1 and MMP-14 mRNA was detected in the endometrium during the estrous cycle and at Day 35 of gestation by qPCR. The expression levels of MMP-1 mRNA was very low (Figure 5C) and statistically insignificant whereas its localization remained below the detection limit at each stage in the cyclic and pregnant endometrium (Figure 7A-E).

Expression of MMP-14 mRNA was significantly higher at after ovulation and decreased during mid-cycle and at Day 35 of gestation; whereas, slight expression was found in the fetal membrane (Figure 5D). MMP-14 mRNA was slightly localized in stroma before ovulation (Figure 7F) but was strongly localized in the stroma after ovulation (Figure 7G) with moderate expression seen during the MEC (Figure 7H). At Day 19 of gestation, MMP-14 mRNA expression was slight in the stroma and luminal epithelium (Figure 7I) but moderately expressed at the site of placentation on the Day 30 of gestation (Figure 7J). The localization of MMP-1 and MMP-14 mRNA has summarized in Table 4.

## Discussion

The purposes of this study are following: 1) to determine the expression of EMMPRIN in bovine endometrium during estrous cycle and early gestation; 2) to find the localization of its expressive cells; and 3) to explore its roles in the endometrial remodeling for the receptivity of embryos in cow. We used a quantitative gene analysis, in situ hybridization and immunohistochemical methods. We examined the expression of MMP-1, -2, -9 and -14 as the indicators for assuming EMMPRIN roles in the remodeling of endometrium in cow. The present study reveals the first sequential evidence that EMMPRIN expresses in bovine endometrium during estrous cycle and the critical period of implantation. The expression of EMMPRIN in the bovine endometrium matched roughly but not completely with that highlighted by the previous studies of humans and rodents. In human, EMMPRIN is highly associated with endometrial remodeling during the menstrual cycle, and it was detected in the luminal and glandular epithelium and stroma [35,36]. In mice, EMMPRIN was expressed in the epithelium around implantation but defects in EMMPRIN expression caused reproductive failure in KO mice [48]. These results strongly suggest that EMMPRIN involves in the process of receptivity in any species like cow, human and rodents. The expression of bovine EMMPRIN during the estrous cycle may be closely related to steroid hormone levels in bovines as in humans and mice [35,48,49]. During the estrous cycle, EMMPRIN expression was found according to cyclic changes in endometrium and was at highest intensity during the middle of estrous cycle when the circulatory progesterone level is in peak, reflecting the regulatory role of progesterone over EMMPRIN as in human [35]. However, estrogenic effects cannot be neglected in cow because expression was found during the estrogenic phase as seen in rodents [17,37].

In the present study, around peri-implantation period, expression of EMMPRIN in luminal epithelial was decreased compared to that observed in the middle of the estrous cycle but ample EMMPRIN was expressed by the peri-attachment conceptus. Active embryonic and endometrial expressions around implantation have been reported in mice [17,48]. The reasons for decreased in epithelial expression of EMMPRIN during this critical period remained unclear. EMMPRIN also has the potential for inducing the ECM degradation necessary for biological functions by itself or by activating neighboring cells to induce MMP production [34]. In the present study, *in situ *hybridization and immunohistochemistry analysis using tissue materials during peri-implantation period showed that conceptus expressed EMMPRIN gene and protein, and MMPs (MMP-2 and -14) were detected in epithelia and subepithelial stromal area. These data suggest that EMMPRIN induces MMP production during this critical period in bovine at feto-maternal interface.

Another important role of EMMPRIN during pregnancy may be maintaining of glandular functions, because the ruminant intercaruncular endometrium contains a large number of uterine glands that synthesize and secrete or transport a variety of substances necessary for the survival and development of the conceptus [50,51]. During the early stage of the estrous cycle, the uterine glands are elongated and coiled and then gradually proliferate and become swollen in order to secrete uterine milk protein [52]. EMMPRIN expression was well correlated with glandular development during the estrous cycle. At Day 19 of gestation, intense staining of EMMPRIN was found on the stroma surrounding the glands. This stromal expression might be from the glandular epithelium because EMMPRIN can shed from the surface of epithelial cells and acts as a diffusible factor, reaching other cells in vicinity [53]. This epithelio-stromal interaction may leads to stimulation of MMPs in lower stromal compartment which further activates other cytokines and growth factors necessary for implantation.

EMMPRIN has been characterized as an important inducer of MMP in tumor tissues [20,54], but has no effects on their physiological inhibitors (tissue inhibitor for MMP-1 and -2; TIMP-1 or -2), therefore modifying the balance among MMP production and activation. Membrane type 1-MMP (MT1-MMP as described as MMP-14 in the present study) cleaves the functional N-terminal domain of EMMPRIN from the cell surface, which is expected to down-regulate its function, but at the same time, the released 22-kDa fragment may mediate the expression of MMP-2 in tumor tissues [55]. In the present study, we detected some different sized EMMPRIN proteins in the endometrial tissues; MEC and three cultured cells (epithelial cells, stromal cells, and trophoblast) by Western blotting as shown in Figure 3, suggesting that the various sizes of EMMPRIN proteins expressed by bovine endometrial cells may correspond to the secretory and membrane-binding forms [43,55]. Therefore, the functions and regulation of EMMPRIN activity during remodeling of the normal endometrium are still difficult to explain because various factors such as membrane-type MMP, tissue inhibitor of metalloproteinases, ADAMTS, etc., are involved [14,55]. Despite significant research into the induction of MMP by EMMPRIN, many fundamental questions persist as to whether the induction of MMP by EMMPRIN is species specific, tissue specific, or related to the functional status of tissues. Few possible explanations have been given by some researchers. EMMPRIN has expressed at all stages in pre-implantation developing embryos but none of MMPs, suggests that EMMPRIN may not function as an MMP inducer in embryos [56]. In a recent report on adult mammary gland development, EMMPRIN did not stimulate the induction of MMP [57]. On the other hand, co-localization of MMP-1 and EMMPRIN was detected in the endometrium during the menstrual cycle [35], which was not seen in current study. The stimulation of MMP production by EMMPRIN via fibroblasts was also shown previously [54]. Uses of recombinant EMMPRIN has been shown to increase the expression of various MMPs in the physiological tissues in dose and time dependent manner, however it's difficult to explain the mechanism of such induction.

To show the possibility of EMMPRIN as a player of MMPs, we examined the expression of MMP-1, -2, -9 and -14 in the cyclic and pregnant endometrium. EMMPRIN was primarily expressed on epithelial cells whereas MMP-2 and -14 were mainly expressed in stromal cells adjacent to basement membrane. Therefore, we have expected that EMMPRIN from the epithelial cells may activate MMP-14, further this MMP-14 activates MMP-2 by binding with TIMP or EMMPRIN may directly activate MMP-2 through its fragment. The mechanism of such activations is still not clearly understood, however some theories have been proposed during the tumor metastasis [58]. EMMPRIN exerts its effect through direct cell-cell interaction in the tumor metastasis by stimulating MMP production from nearby fibroblasts [59]. It has been suggested that EMMPRIN serves as its own counter-receptor in cancer cells thus stimulating MMP production through homophilic interaction between EMMPRIN molecules on opposing cells [60]. This hypothesis has been investigated in tumor cell progression but the situation in normal cells remains unclear. Further studies are needed to clarify the regulatory role of EMMPRIN in endometrial remodeling as a means of regulating the implantation process in non-invasive implantation animals; ruminant.

## Conclusions

In this study, EMMPRIN was differentially expressed in endometrium including on feto-maternal interfaces. Expression of EMMPRIN on feto-maternal interfaces may facilitate adhesion and fusion of embryo to luminal epithelium by directly itself through physiological tissues remodeling and developmental process, and/or stimulating MMPs to compensate endometrial functions.

## List of abbreviations

EMMPRIN: extracellular matrix metalloproteinase inducer; MMP: matrix metalloproteinase; ECM: extracellular matrix; ADAMTS: a disintegrin and metalloprotease with thrombospondin motif; qPCR: quantitative RT-PCR; AOV: stage after ovulation; MEC: stage middle of estrous cycle; BOV: stage before ovulation; DIG: Digoxigenin; BES: bovine endometrial stromal cells; BEE: bovine endometrial epithelial cells; BT-1: bovine trophoblastic cell; MT1-MMP: Membrane type 1-MMP;

## Competing interests

The authors declare that they have no competing interests.

## Authors' contributions

BM participated in the design of study, carried out the experiments and wrote the manuscript. K. Koshi, KU and MH helped in histological study. K. Kizaki, TT helped in collection of sample and guided in experimental procedures. KH participated in the design and coordination of study and helped to draft the manuscript. TS and AT prepared the rabbit anti-EMMPRIN antibody and helped in Western blot analysis. All authors read and approved the final manuscript for publication.
